# Prevention of Diseases of the Skin

**Published:** 1858-04

**Authors:** 


### THE PREVENTION OF DISEASES OF THE SKIN.*

Mr. Hunt's little handbook commends itself specially to the sanitary student. His concluding chapter is a short but excellent epitome of the general means for the prevention of diseases of the skin.

Speaking of bathing, Mr. Hunt thinks, with due precautions, that either the warm or the cold bath may be employed with equal success. Speaking of air and exercise, he thus writes:

"*Air and Exercise.* Those diseases of the skin, the cure of which is found most tedious and difficult, generally occur in the sedentary class. Shoemakers, tailors, weavers, sempstresses, and artizans of all kinds who work many hours per day in close, confined apartments, often suffer severely, both from general weakness and impaired health, as also from various cutaneous maladies arising from breathing impure air. Yet the remedy is much in their own hands. Most of them reside within easy walking distance of some of the now numerous parks provided for their health. Early in the morning, in the summer especially, for one or two hours before the day's work commences, the air of these parks is as pure and salubrious, and as sweet with the exhalations of leaves

* A Guide to the Treatment of Diseases of the Skin. By THOMAS HUNT, F.R.C.S. London: Churchill. 1857.

and flowers as the most distant country park. A walk of three or four miles before breakfast, when once it becomes a habit, gives tone and strength and spirits for the sedentary toil of the day, in a degree incredible to those who never imbibe the sweet breath of morn. Yet the Regent's Park, accessible to tens of thousands, can scarcely count its early visitors by scores; and Primrose Hill is seldom crowned by more than a dozen strollers before the smoke from ten thousand chimneys obscures the view and pollutes the air. I do not hesitate to say that by the morning walk and evening bath a large proportion of the skin diseases of the sedentary might be prevented, the general health of all invigorated, and the mortuary returns of the Registrar-General reduced more sensibly than by all the excellent sanitary improvements now in progress. The early-closing movement may be turned to good account by an evening stroll in the parks; but, as compared with the *early-rising movement*, it is unimportant.

"The morning stroll has other recommendations equally strong, which it may not be deemed an unpardonable digression to mention; I refer to its moral influence. The quiet of the morning invites, almost compels, calm meditation. To the young it offers no temptations. Places of questionable or immoral amusement are closed. The profligate, vicious, and profane are, for the most part asleep, and for a time innocuous and inoffensive; the morning air often exhilarates, always refreshes and cheers the spirits. A gleam of hope, and a sense of peace and tranquillity, if not of contentment and happiness, may beam on the mind, long depressed and fretful, almost as naturally on these occasions as the rays of the rising sun enliven and adorn the scenes of nature."

Speaking of diet, Mr. Hunt tells us that he knows of no articles of diet which ought to be avoided in all skin diseases. As a general rule, he "holds it to be a presumption to prescribe any particular form of diet." The instinct will prove the best guide to the repast.

